# Addressing the gap in preterm resuscitation practices in high-income and low-middle income countries: a multicenter survey of the Asian neonatal network collaboration

**DOI:** 10.3389/fped.2024.1517843

**Published:** 2025-01-30

**Authors:** Rinawati Rohsiswatmo, Rizalya Dewi, Jennie Sutantio, Zubair Amin, Young-Ah Youn, Sae Yun Kim, Su Jin Cho, Yun Sil Chang, Satoshi Kusuda, Fuyu Miyake, Tetsuya Isayama

**Affiliations:** ^1^ Department of Child Health, Universitas Indonesia/Dr. Cipto Mangunkusumo Hospital, Jakarta, Indonesia; ^2^ Department of Child Health, Budhi Mulia Mother and Child Hospital, Pekanbaru, Indonesia; ^3^ Department of Neonatology, Khoo Teck Puat-National University Children’s Medical Institute, National University Hospital, Singapore, Singapore; ^4^ Department of Pediatrics, College of Medicine, The Catholic University of Korea, Seoul, Republic of Korea; ^5^ Department of Pediatrics, Ewha Womans University College of Medicine, Seoul, Republic of Korea; ^6^ Department of Pediatrics, Samsung Medical Center, Sungkyunkwan University School of Medicine, Seoul, Republic of Korea; ^7^ Neonatal Research Network of Japan, Kyorin University, Tokyo, Japan; ^8^ Division of Neonatology, National Research Institute for Child Health and Development, Tokyo, Japan

**Keywords:** neonatal resuscitation, gap practice, low-middle income countries, high-income countries, multicenter survey, preterm (birth)

## Abstract

**Background:**

Optimum neonatal resuscitation practices are vital for improving neonatal survival and neurodevelopment outcomes, particularly in extremely preterm infants. However, such practices may vary between high-income countries (HICs) and low-middle-income countries (LMICs). This study aimed to evaluate the resuscitation practices of high-risk infants in a large multi-country sample of healthcare facilities among HICs and LMICs in Asia under the AsianNeo Network.

**Methods:**

In 2021, a customized 6-item online survey on resuscitation practices of infants born at <29 weeks gestation (or birth weight <1,200 g) was sent by the representative of each country's neonatal network to all the Neonatal Intensive Care Units (NICUs) participating in AsianNeo network. At the time of the survey, there were 446 participating hospitals in eight countries: four high-income countries (Japan, Singapore, South Korea, and Taiwan) and four low-middle-income countries (Malaysia, Indonesia, Philippines, and Thailand).

**Results:**

The study included 446 hospitals, with a response rate of 72.6% (ranging from 62.7% to 100%), with 179 (55.2%) in HICs and 145 (44.7%) in LMICs. Routine attendance of experienced NICU physicians during resuscitations is reported to be higher in HICs than LMICs, both during daytime (79% vs. 40%) and nighttime (62% vs. 23%). The NRP guidelines in each country were varied, with 4 out of 8 countries using indigenously developed guidelines. Equipment availability during resuscitation was also variable; saturation monitors, radiant warmers, and plastic wraps were available in almost all hospitals, whereas oxygen and air blenders, heated humidified gas, and end-tidal CO_2_ detectors were more available in HICs. The most common device for Positive Pressure Ventilation (PPV) was the T-piece resuscitator (52.3%).

**Conclusion:**

The neonatal resuscitation practices for extremely preterm infants, encompassing staff, equipment, and guidelines, exhibited variance between HICs and LMICs in the AsianNeo region. Further enhancements are imperative to narrow this gap and optimize neonatal outcomes.

## Background

Despite the significant progress achieved in recent decades and the notable improvement in survival rates, the care of very preterm and extremely preterm infants remains a complex endeavor, particularly from the moment of delivery (1–3). These infants are more prone to necessitate resuscitation and are at a heightened risk of experiencing complications during the resuscitative process (4). Multiple factors contribute to this, including susceptibility to hypothermia, underdeveloped pulmonary function, susceptibility to infection, and increased vulnerability to organ injury (5). Depending on the specific medical circumstances, resuscitative measures may encompass a range of increasingly intensive interventions, from ventilation and oxygenation to the administration of epinephrine and volume expanders (6).

Interventions to improve neonatal resuscitation are essential to any strategy to reduce neonatal mortality. Training neonatal healthcare providers in neonatal resuscitation should result in a 30% reduction in mortality among full-term babies and 5%–10% in preterm babies (7). Besides the personnel's skill and knowledge, adequate equipment in all delivery settings is crucial. Despite this fact, there has been a disparity between different countries regarding improving neonatal resuscitation, especially among LMICs (8). Neonatal mortality rates in HICs range from 0.8 to 2.4 per 1,000 live births, while in LMICs, the rates range from 4.6 to 12.6 per 1,000 live births (9).

Surveys on neonatal resuscitation have been conducted in high-resource countries (10–12) and more recently, in low and middle-income countries (7, 13, 14). However, a multicenter study that emphasizes the baseline characteristics leading to such discord between high-income and low—and middle-income countries has not yet been conducted. This survey aims to describe differences in resuscitation practices and equipment availability in a large representative sample between different Asian countries. We report data regarding the personnel, device, and guideline/program available for neonatal resuscitation, especially for infants born at <29 weeks gestation (or birth weight <1,200 g) in the AsianNeo regions.

## Methods

### Participants

The survey was conducted under the umbrella of the Asian Neonatal Network Collaboration (AsianNeo), which was established in 2019 to improve neonatal care in Asia. The AsianNeo currently consists of nine neonatal networks from Indonesia, Japan (network leader), Malaysia, Philippines, Singapore, South Korea, Taiwan, Thailand, and Sri Lanka (9, 15), but only eight countries participated in the survey (Table 1). The classification into HICs and LMICs is established using the Gross National Income (GNI) per capita, determined through the World Bank Atlas method. Countries with a GNI per capita above US$14,005 are considered high-income, while those below this threshold are classified as LMICs (16). Four high-income countries (Japan, Singapore, South Korea, and Taiwan) and four low-income countries (Malaysia, Indonesia, Philippines, and Thailand).

**Table 1:** Number of participating NICUs according to the country.

Country	Number of surveyed hospitals	Response rate (%)
High-income countries		
Singapore	3	3 (100)
Japan	225	141 (62.7)
Korea	13	12 (92.3)
Taiwan	23	23 (100)
Low-middle income countries		
Philippines	16	13 (81.3)
Malaysia	35	34 (97.1)
Indonesia	38	36 (100)
Thailand	93	62 (66.7)
Total	446	324 (72.6)

### Survey instrument

A survey on the resuscitation of high-risk infants (gestational age <29 weeks or birth weight <1,200 g) was conducted from February to March 2021 at level III neonatal medical centers across Asian countries, including both government and private hospitals. The decision to extend the gestational age and birth weight cutoff beyond extreme preterm and ELBW is due to a limited number of hospitals in certain countries that actively resuscitate infants in those categories. The survey consisted of 6 inquiries concerning the presence of the resuscitation team, the guidelines utilized, the standard device for positive pressure ventilation, the percentage of trained primary resuscitators, and other devices used during resuscitation (Appendix 1).

Primary resuscitators are categorized into four groups: experienced NICU physicians, less-experienced NICU physicians, non-NICU physicians, and midwives/nurses. Experienced NICU physicians are those who have at least 3 years’ experience of full-time work in level-3- NICU (neonatologists, registerers/hospitalists, general pediatricians, etc.), while less-experienced NICU physicians have less than 3 years' experience of full-time work in level-3-NICU (registerer/hospitalists, general pediatricians, NICU fellows, pediatric residents in NICU rotation, etc.). Non-NICU physicians who do not belong to NICUs (e.g., general pediatricians, non-pediatric physicians). Midwives and nurses are combined in one group because in some countries, including Indonesia, they receive similar training in neonatal resuscitation.

The survey was performed using an English-language structured online questionnaire (SurveyMonkey®) and completed by the person or his/her designate in charge of neonatal care at each hospital. The questionnaire was translated into the native language of non-English-speaking countries. The country representative of each neonatal network sent the request for the survey to all the NICUs participating in the AsianNeo.

### Statistical analysis

We performed statistical analyses using STATA 14.0 (STATA Corp, College Station, TX, USA). We conducted proper data coding and categorization and verified the data for completeness and accuracy. We performed descriptive statistics for personnel and program variables and obtained the means or medians for device variables. Categorical data were expressed as numbers and percentages.

### Ethics

The ethical approval was obtained from the Ethics Committee of the Faculty of Medicine, University of Indonesia, Cipto Mengunkusumo Hospital, Jakarta, Indonesia. All participants provided informed consent to participate in this study.

## Results

The study involved 446 hospitals, with a response rate of 72.6%, varying from 62.7% to 100% across different countries. The distribution of participating hospitals was equal between HICs and LMICs, with 179 (55.2%) located in HICs and 145 (44.7%) in LMICs (Table 1). Data for neonatal resuscitation personnel, equipment, and trained staff were missing from 5 hospitals in Japan (3), Malaysia (1), and Taiwan (1).

### Resuscitation team during daytime and nighttime

**Figure 1:**
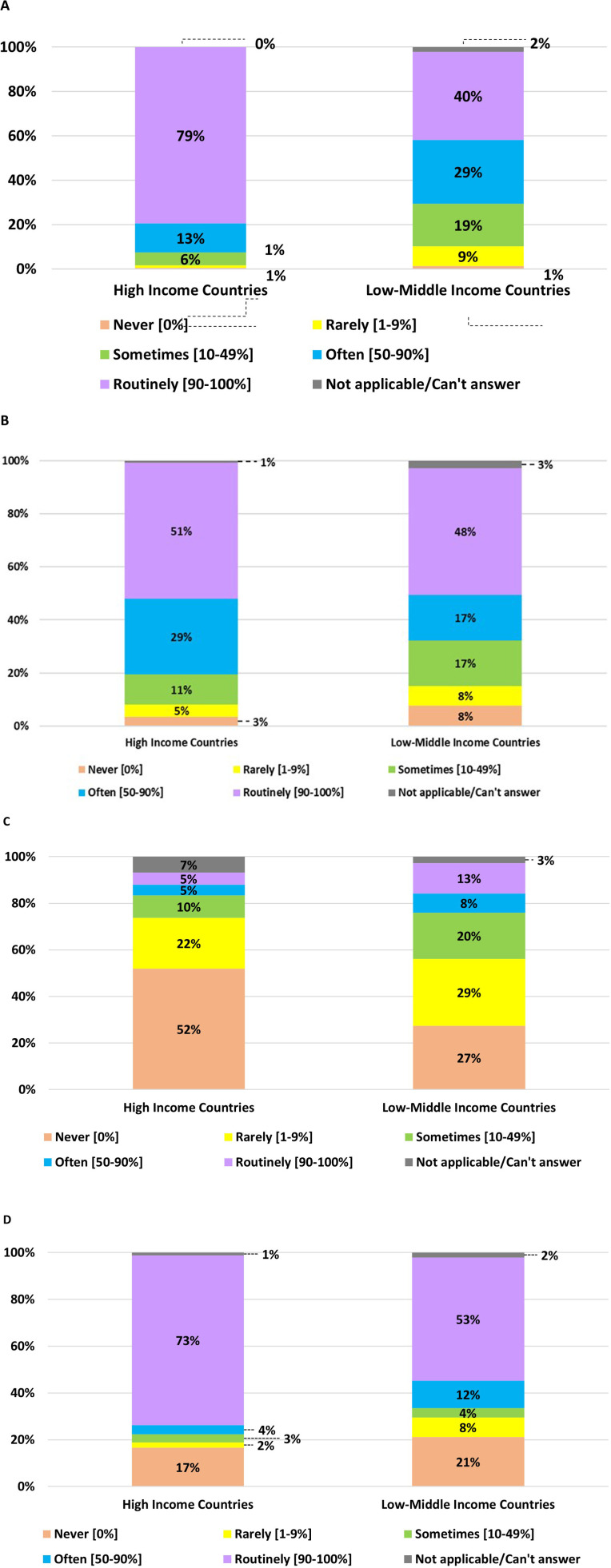
Personnel attending the resuscitation of infants born at <29 weeks gestation (or birth weight <1,200 g) during daytime in HICs vs. LMICs. **(A)** Experienced NICU physicians with ≥3 years of work experience. **(B)** Less-experienced NICU physicians with <3 years of work experience. **(C)** Non-NICU physicians. **(D)** Midwives or nurses.

The personnel attending neonatal resuscitation varies during the daytime in different countries (Appendix 2) and among the HICs vs. LMICs (Figure 1). HICs have a higher presence of experienced and midwives or nurses during the daytime than those in LMICs (Figures 1A,D). However, similarities in resuscitation team components between the two groups are observed in the routine attendance of less experienced NICU physicians and non-NICU physicians (Figures 1B,C).

**Figure 2:**
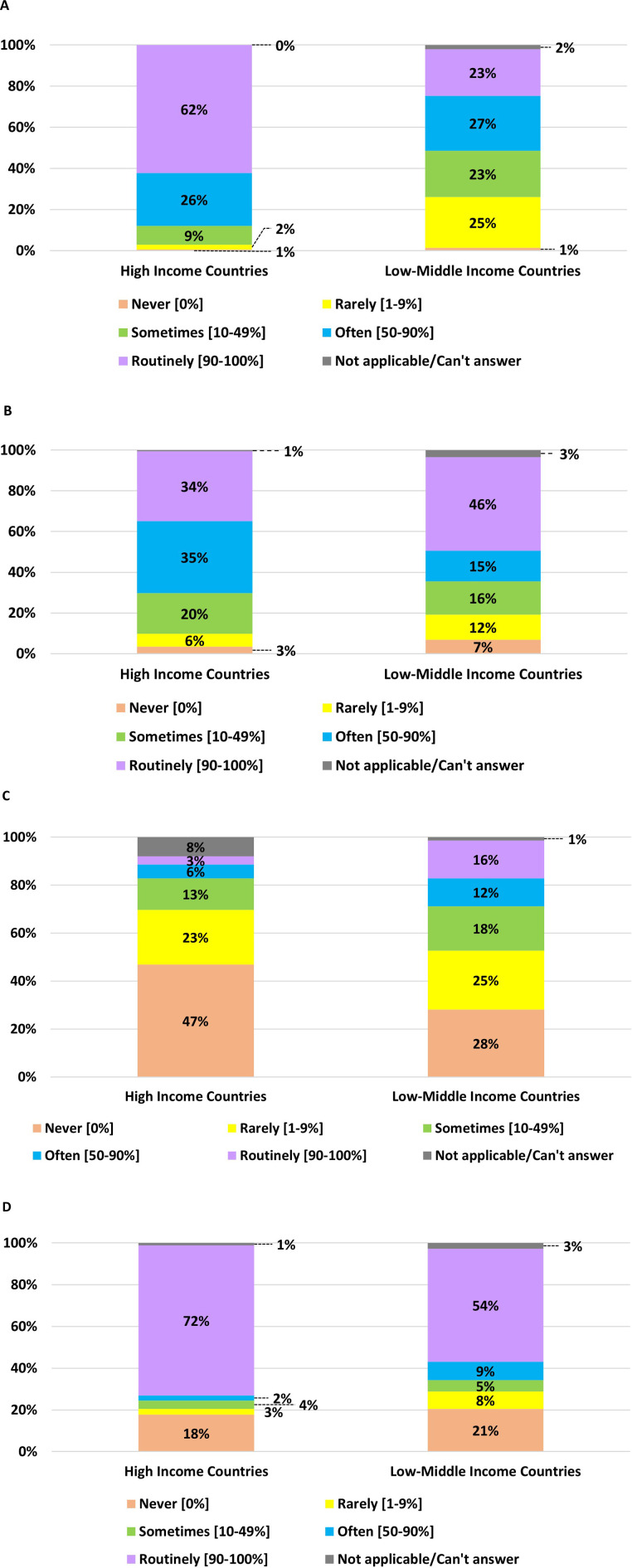
Personnel attend the resuscitation of infants born at <29 weeks gestation (or birth weight <1,200 g) during nighttime in HICs vs. LMICs. **(A)** Experienced NICU physicians with ≥3 years of work experience. **(B)** Less-experienced NICU physicians with <3 years of work experience. **(C)** Non-NICU physicians. **(D)** Midwives or nurses.

Figure 2 shows the team present for nighttime neonatal resuscitation in HICs and LMICs. The details of the nighttime team across countries are presented in Appendix 2. Compared to daytime resuscitation, the presence of experienced NICU physicians during nighttime has decreased overall in all participants, from 61.9% to 44%. However, it is still more common in HICs than LMICs. Attendance among less-experienced NICU physicians is lower during night shifts compared to LMICs. During nighttime resuscitation, the proportion of less experienced NICU and non-NICU physicians, midwives, or nurses is comparable to daytime resuscitation.

Most participants in MICs and LMICs had a SpO2 monitor, radiant warmers, plastic bags or wraps, and mechanical suctioning equipment. Nevertheless, there was a substantial discrepancy between HICs and LMICs regarding the availability of some essential equipment, including blenders of air and oxygen, ECG monitors, and end-tidal CO_2_ monitors. Interestingly, gas humidifiers were slightly more prevalent in LMICs than in HICs (Table 2). Details of equipment availability in each country is presented in Appendix 3.

**Table 2:** Device or equipment for neonatal resuscitation between HICs and LMICs.

Device	Low-middle income countries (*n* = 147)		High income countries (*n* = 179)	
	*n*	%	*n*	%
Blender of air and oxygen	103	70.07	170	94.97
Gas humidifier	77	52.38	81	45.25
ECG monitor	40	27.21	143	79.89
SpO_2_ monitor	144	97.96	175	97.77
End-tidal CO_2_ monitor	5	3.40	99	55.31
Radiant warmer	144	97.96	173	96.65
Plastic bags or plastic wraps	143	97.28	165	92.18
Mechanical suctioning equipment	141	95.92	172	96.09

**Figure 3:**
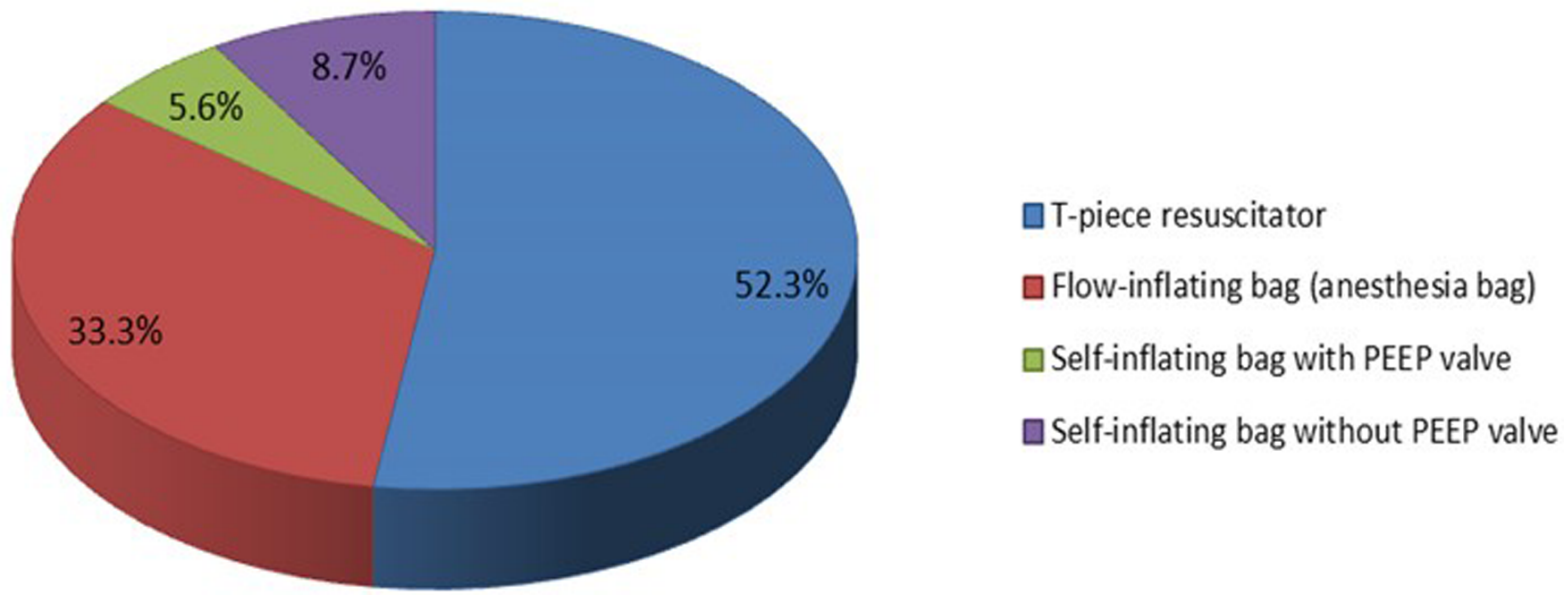
The most common device for positive pressure ventilation (PPV) used in neonatal resuscitation of very preterm infants born at <29 weeks gestation (or birth weight <1,200 g) immediately after birth.

The equipment used for providing respiratory support during resuscitation varied. However, the T-piece resuscitator was the most used for very preterm infants (Figure 3). Interestingly, the T-piece resuscitator is more widely used in low- and middle-income countries (LMICs) than in high-income countries (HICs) (Table 3). Japan is the country with the lowest utilization of the T-piece resuscitator and instead prefers the use of the flow-inflating bag (Appendix 4).

**Table 3:** The most common device for positive pressure ventilation (PPV) used in neonatal resuscitation of very preterm infants born at <29 weeks gestation (or birth weight <1,200 g) just after birth.

Device	High income countries (*n* = 147)		Low-middle income countries (*n* = 179)	
	*n*	%	*n*	%
T-piece resuscitator	39	22	126	88
Flow-inflating bag (anesthesia bag)	107	61	0	0
Self-inflating bag with PEEP valve	12	6.8	6	4.1
Self-inflating bag without PEEP valve	16	9.1	11	7.7

**Figure 4:**
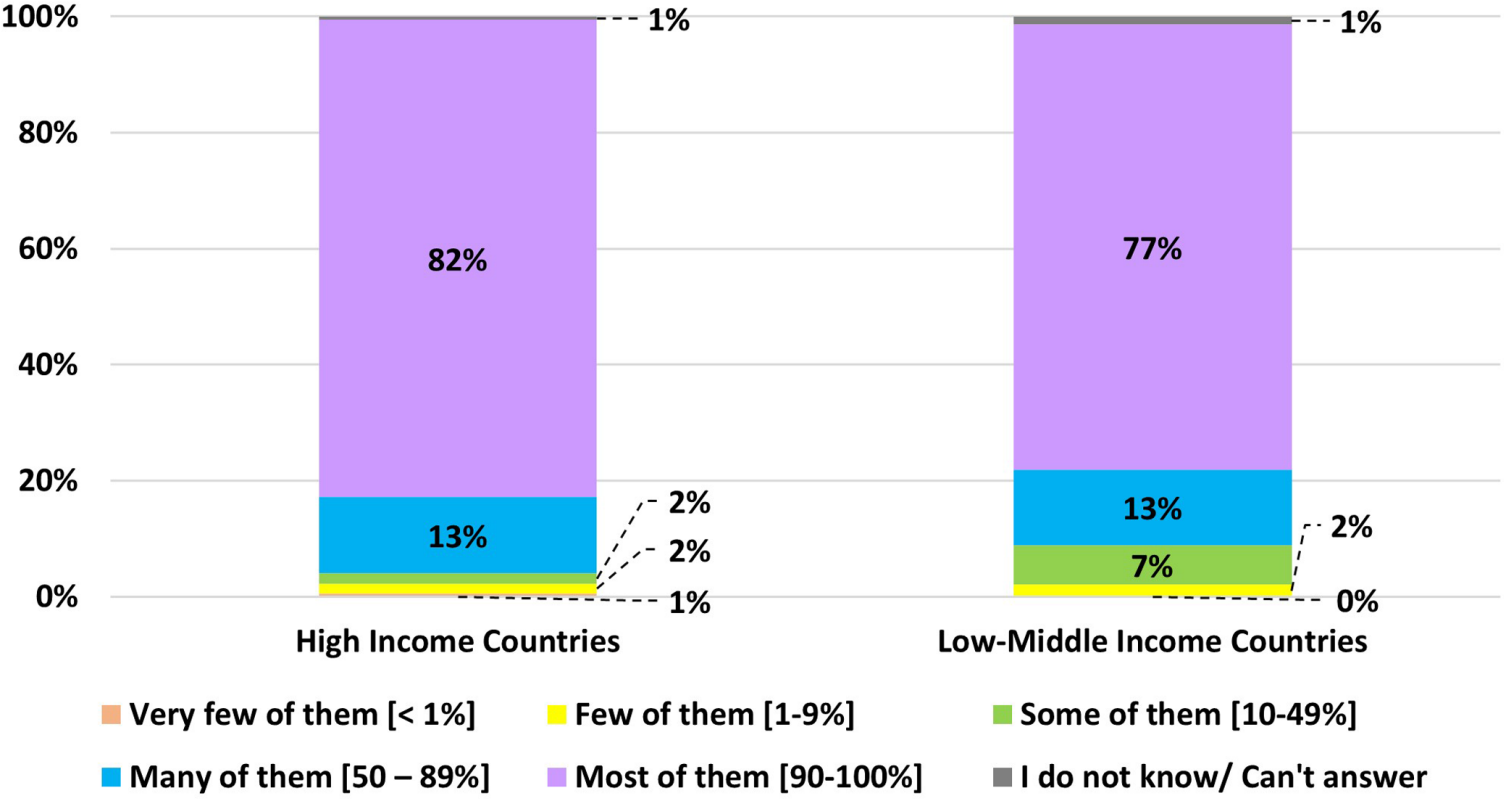
The proportion of the main resuscitators of very preterm infants (gestational age <29 weeks or birth weight <1,200 g) trained or certified for neonatal resuscitation programs regardless of the type of the program.

Table 4 presents the variations in resuscitation guidelines in Asian-Neo countries. However, American Academy of Pediatrics (AAP) guidelines are used in half of the participating countries, both in HICs and LMICs. Most primary resuscitators already have either certification or training in neonatal resuscitation programs, both in HICs and LMICs (Figure 4).

**Table 4:** The resuscitation guideline/program.

Country	Neonatal resuscitation program (NRP)
High-income countries	
Singapore	Singapore NRP
Japan	Japanese NRP
Korea	AAP NRP
Taiwan	AAP NRP (modified)
Low-middle income countries	
Philippines	Philippine Essential Newborn Care & Resuscitation Program (NRPh+)
Malaysia	AAP NRP
Indonesia	Indonesian NRP
Thailand	AAP NRP

AAP, American Academy of Pediatrics.

## Discussion

This survey provides the first comprehensive analysis of neonatal resuscitation preparedness and resources in Asian countries. It highlights disparities in resuscitation practices for infants born before 29 weeks of gestational age and weighing less than 1,200 g, comparing HICs to LMICs within the AsianNeo network. Although we do not examine the differences in neonatal mortality rates between these two groups in this survey, a previously published AsianNeo report indicated that neonatal mortality rates in HICs range from 0.8 to 2.4 per 1,000 live births. In contrast, in LMICs, the rates range from 4.6 to 12.6 per 1,000 live births (9). These disparities may stem from differences in personnel and medical equipment availability between the two groups.

The key factor affecting neonatal outcomes in both groups is the availability of experienced NICU physicians, which is more common in high-income countries (HICs) than in low- and middle-income countries (LMICs), both during the day and at night. Although expected, this matter warrants attention as per the recommendation of the American Academy of Pediatrics, which stipulates the presence of a proficient neonatal resuscitation team. In the event of an anticipated high-risk birth, such as that of an extremely preterm infant, it is imperative to assemble a well-staffed team capable of providing positive pressure ventilation, tracheal intubation, chest compressions, emergency vascular access, medication preparation, and event documentation before the birth (5). Based on the meta-analysis conducted by Patel et al. (8), implementation of neonatal resuscitation training resulted in a significant reduction in early neonatal mortality.

The second most significant factor impacting outcomes is the equipment used in delivery or resuscitation rooms for extremely preterm infants. It was noted that LMICs lacked oxygen and air blenders, ECG monitors, and end-tidal CO_2_ (EtCO_2_) monitors. The American Heart Association's current recommendations advise adjusting the delivery of oxygen to newborns to achieve specific target oxygen levels rather than simply administering 100% oxygen (17). Without consistent access to oxygen blending, newborns in low-resource environments are often treated with pure oxygen and are placed at significant risk of hyperoxia-induced injury (18). For example, in India, the incidence of ROP is markedly higher than in high-income countries, and ROP has become the leading cause of avoidable blindness in children (19). A cardiac monitor with 3 chest leads or limb leads provides a rapid and reliable method of continuously displaying the baby's heart rate if the pulse oximeter has difficulty acquiring a signal (5). The International Consensus on Cardiopulmonary Resuscitation and Emergency Cardiovascular Care Science with Treatment Recommendations (CoSTR) also echoed this (20). Detecting exhaled CO_2_ and an increasing heart rate are the primary methods of confirming endotracheal tube insertion (5). EtCO_2_ monitoring may be an early indication of lung expansion and may help to guide successful respiratory support in the delivery room—an observational study conducted by Hawkes et al. (21) reported that EtCO_2_ monitoring in the delivery room is feasible and safe. EtCO_2_ values obtained after birth reflect the establishment of functional residual capacity and effective ventilation. Other clinical practices to confirm correct endotracheal tube placement include condensation in the endotracheal tube, chest movement, and the existence of equal breath sounds bilaterally. However, these practices have not been systematically evaluated in newborns (22).

There is room for improvement in resuscitation equipment across all participants. The gas humidifier was not universally available in most resuscitation rooms in LMICs and HICs. Oxygen given to newborns for a prolonged period of time should be heated and humidified to prevent heat loss (5). A meta-analysis conducted by Meyer et al. (23). The use of heated humidified gases showed that the number of infants with severe hypothermia (<35.5°C) was significantly reduced. It showed that a gas humidifier improved admission temperature in preterm infants.

From the survey, it was noteworthy that various resuscitation guidelines/programs were used in the participating countries, with 4 out of 8 countries using indigenously developed guidelines and other countries using the AAP guidelines. There are some differences among the national guidelines. For example, oxygen administration is divided into ≥35 weeks (21%) and <35 weeks (21%–30%) gestational age in both Indonesia and Japan guidelines, while Singapore guideline uses different categories such as ≥33 weeks (21%) and <33 weeks (21%–30%) gestational age. The room temperature is set into 23°C–25°C in both Singapore and Japan guidelines, while Indonesia guideline uses 24°C–26°C (16–18).

The strength of this study is representative data from both low-middle income countries and high-resource countries, comprehensive coverage of the survey's participants across the countries with high response rates even in the pandemic era with otherwise limited access to conduct direct surveys. However, this study has some limitations, such as the different number of participants in each country and reliance on self-reporting by a person contacted through the survey without inspection of the sites. This would cause room for a degree of bias; however, with the limitation of direct surveys, this is a viable alternative and room for possible future improvement of multicenter surveys. Another limitation we can improve in future studies is the details of each participant's hospital, resuscitation team size, and area background regarding the socio-economic and geographical conditions. These details could aid in a more specific strategy to improve neonatal resuscitation practice to the potential of each hospital.

## Conclusion

We identified significant variations in neonatal resuscitation practices among Asian countries among the AsianNeo collaborators. This survey identifies the resources available, such as personnel, equipment, and program/guidelines, for neonatal resuscitation in low-income to high-resource countries with various socio-economic backgrounds. Our study provides the possibility for improvements through the AsianNeo network in neonatal care with specific strategies, using background data of each hospital as a basis.

## Data Availability

The original contributions presented in the study are included in the article/Supplementary Material, further inquiries can be directed to the corresponding author.

## Appendix 1

AsianNeo survey: delivery and resuscitation.

1. During daytime, how often the following personnel attend the resuscitation of infants born at <29 weeks gestation (or birth weight <1,200 g) in your hospitals? (The attendance at resuscitation includes not only those as a main resuscitator but also those as a support person for neonatal resuscitation).

**Table:**

	Routinely (90%–100%)	Often (50%–90%)	Sometimes (10%–49%)	Rarely (1%–9%)	Never (0%)	Not applicable can't answer
Experienced NICU physicians who have ≥3 years’ experience of full-time work in level-3- NICU (neonatologists, registerers/hospitalists, general pediatricians, etc.)						
Less-experienced NICU physicians who have <3 years’ experience of full-time work in level-3-NICU (registerer/hospitalists, general pediatricians, NICU fellows, pediatric residents in NICU rotation, etc.)						
Non-NICU physicians who do not belong to NICUs (e.g., general pediatricians, non-pediatric physicians)						
Midwives or nurses						

2. During nighttime, how often the following personnel attend the resuscitation of infants born at <29 weeks gestation (or birth weight <1,200 g) in your hospitals? (The attendance at resuscitation includes not only those as a main resuscitator but also those as a support person for neonatal resuscitation).

**Table:**

	Routinely (90%–100%)	Often (50%–90%)	Sometimes (10%–49%)	Rarely (1%–9%)	Never (0%)	Not applicable can't answer
Experienced NICU physicians who have ≥3 years’ experience of full-time work in level-3- NICU (neonatologists, registerers/hospitalists, general pediatricians, etc.)						
Less-experienced NICU physicians who have <3 years’ experience of full-time work in level-3-NICU (registerer/hospitalists, general pediatricians, NICU fellows, pediatric residents in NICU rotation, etc.)						
Non-NICU physicians who do not belong to NICUs (e.g., general pediatricians, non[1]pediatric physicians)						
Midwives or nurses						

3. What is the most common device for positive pressure ventilation used in neonatal resuscitation of very preterm infants born at <29 weeks gestation (or birth weight <1,200 g) just after birth in your hospital?
  a. T-piece resuscitator
  b. Flow-inflating bag (anesthesia bag)
  c. Self-inflating bag with PEEP valve
  d. Self-inflating bag without PEEP valve
  e. Other (please specify)
4. What is the most common device for positive pressure ventilation used in neonatal resuscitation of very preterm infants born at <29 weeks gestation (or birth weight <1,200 g) just after birth in your hospital?
  a. T-piece resuscitator
  b. Flow-inflating bag (anesthesia bag)
  c. Self-inflating bag with PEEP valve
  d. Self-inflating bag without PEEP valve
  e. Other (please specify)
5. How much proportion of the main resuscitators* of very preterm infants (gestational age <29 weeks or birth weight <1,200 g) are trained or certified for neonatal resuscitation program regardless of the type of the program? Main resuscitators are the persons who manage respiratory support in resuscitation such as bag-mask ventilation.
  a. Most of them [90%–100%]
  b. Many of them [50%–89%]
  c. Some of them [10%–49%]
  d. Few of them [1%–9%]
  e. Very few of them [<1%]
  f. I do not know can't answer
6. Does your NICU generally use the following device or equipment for neonatal resuscitation of very preterm infants born at <29 weeks gestation (or birth weight <1,200 g) in delivery or resuscitation rooms when needed? Please select YES or NO for each device or equipment.

**Table:**

	Yes	No	Can't answer
Blender of air and oxygen (mixed gas of air and oxygen)			
Gas humidifier (to humidify the air or oxygen for respiratory support)			
ECG monitor (Electrocardiogram)			
SpO2 monitor (oxygen saturation monitor)			
End-tidal CO_2_ detector (to check endotracheal intubation)			
Radiant warmer (to warm newborn infants)			
Plastic bag or plastic wraps (to keep body temperature of very preterm infants)			
Mechanical suctioning equipment to generate negative pressure (Not a bulb syringe)			

## Appendix 2

Personnel attending the resuscitation during daytime and nighttime across countries.

Daytime attendance

**Table:**

	Singapore				Japan				South Korea				Taiwan			
	EP	LEP	NP	MN	EP	LEP	NP	MN	EP	LEP	NP	MN	EP	LEP	NP	MN
Never [0]			67		0.7	4.3	54	17			67	25			32	14
Rarely [1–9]						5.8	22	2.9	8.3		8.3		4.5		27	
Sometimes [10–49]					2.2	11	8	1.4	25		8.3	8.3	18	23	23	14
Often [50–90]					9.4	34	5.1	2.9	50			17	18	14	4.50	5
Routinely [90–100]	100	100	33	100	88	44	2.9	75	17	100	8.3	50	59	64	14	68
Not applicable/can't answer						0.7	8	1.4			8.3					
	Philippines				Malaysia				Indonesia				Thailand			
	EP	LEP	NP	MN	EP	LEP	NP	MN	EP	LEP	NP	MN	EP	LEP	NP	MN
Never [0]		7.7	31	7.7	3		21	18	2.8	8.3	25			11	31	32
Rarely [1–9]	7.7		23		21	6.1	39	12	2.8	2.8	25		6.5	13	29	10
Sometimes [10–49]	46		15		30	9	21		5.6	25	31		16	21	13	4.8
Often [50–90]	23		7.7	23	50	27	6.10	21	31	17	8.30		29	15	10	6.5
Routinely [90–100]	23	92	15	69	17	55	12	48	56	47	11	100	47	35	13	42
Not applicable/can't answer			7.7			3			2.8				1.6	4.8	4.8	4.8

EP, experienced physician; LEP, less-experienced physician; NP, non-NICU physician; MN, midwives nurses.

Nighttime attendance

**Table:**

	Singapore				Japan				South Korea				Taiwan			
	EP	LEP	NP	MN	EP	LEP	NP	MN	EP	LEP	NP	MN	EP	LEP	NP	MN
Never [0]					0.7	4.3	51	17			50	33			27	14
Rarely [1–9]						7.2	22	2.2	17		17	17	9	4.5	32	
Sometimes [10–49]					3.6	22	13	2.9	25		8.3		36	23	18	14
Often [50–90]	100				22	42	5.8	2.2	42		8.3		27	18	4.5	4.5
Routinely [90–100]		100	33	100	73	24	0.7	74	17	100	8.3	50	27	55	14	68
Not applicable/can't answer			67			0.7	7.2	1.4			8.3				4.5	
	Philippines				Malaysia				Indonesia				Thailand			
	EP	LEP	NP	MN	EP	LEP	NP	MN	EP	LEP	NP	MN	EP	LEP	NP	MN
Never [0]		7.7	38	7.7	3		18	18	2.8	8.3	31			9.7	29	29
Rarely [1–9]	31		7.7		55	15	42	12	8.3	14	22		17.7	13	23	11
Sometimes [10–49]	38		15		24	9.1	12	3	25	17	22		16	24	19	8.1
Often [50–90]	15	7.7	15	23	6.1	21	15	12	31	17	11		39	11	10	3.2
Routinely [90–100]	15	85	15	69	9	52	12	55	31	44	14	100	26	35	18	42
Not applicable/can't answer			7.7		3	3			2.8				1.6	6.5	1.6	6.5

EP, experienced physician; LEP, less-experienced physician; NP, non-NICU physician; MN, midwives nurses.

## Appendix 3

Equipment for neonatal resuscitation across countries.

**Table:**

Device	High-income countries (%)				Low-middle-income countries (%)			
	Singapore (*n* = 3)	Japan (*n* = 141)	Korea (*n* = 12)	Taiwan (*n* = 23)	Philippines (*n* = 13)	Malaysia (*n* = 34)	Indonesia (*n* = 36)	Thailand (*n* = 62)
Blender of air and oxygen	100	98	100	86	53	69	91	61
Gas humidifier	33	46	50	45	69	63	0	43
ECG monitor	0	81	75	95	30	27	0	22
SpO_2_ monitor	100	100	100	100	100	93	100	100
End-tidal CO_2_ Monitor	100	65	0	27	0	0	8.3	3.2
Radiant warmer	100	99	100	100	100	100	100	98
Plastic bags or plastic wraps	100	92	100	100	92	100	100	98
Mechanical suctioning equipment	100	97	100	100	100	93	97	96

## Appendix 4

Most common device for positive pressure ventilation (PPV) across countries.

**Table:**

	High-income countries *n* (%)				Low-middle-income countries *n* (%)			
	Singapore	Japan	Korea	Taiwan	Philippines	Malaysia	Indonesia	Thailand
T-piece resuscitator	3 (100)	13 (9.4)	12 (100)	11 (50)	3 (23)	32 (97)	35 (9)	56 (90)
Flow-inflating bag (anesthesia bag)	0	106 (76)	0	1 (4.5)	0	0	0	0
Self-inflating bag with PEEP valve	0	8 (5.8)	0	4 (18)	1 (7.7)	0	1 (2.8)	4 (6.5)
Self-inflating bag without PEEP valve	0	11 (8)	0	5 (22)	8 (61)	1 (3)	0	2 (3.2)
